# Misuse of Prescription Opioid Medication among Women: A Scoping Review

**DOI:** 10.1155/2016/1754195

**Published:** 2016-04-17

**Authors:** Natalie Hemsing, Lorraine Greaves, Nancy Poole, Rose Schmidt

**Affiliations:** British Columbia Centre of Excellence for Women's Health, Vancouver, BC, Canada V6H 3N1

## Abstract

*Background*. National data from Canada and the United States identify women to be at greater risk than men for the misuse of prescription opioid medications. Various sex- and gender-based factors and patient and physician practices may affect women's use and misuse of prescription opioid drugs.* Objectives*. To explore the particular risks, issues, and treatment considerations for prescription opioid misuse among women who experience chronic noncancer pain and trauma.* Methods*. A scoping review for articles published between January 1990 and May 2014 was conducted on sex- and gender-based risks and treatment considerations among women who experience chronic noncancer pain and trauma.* Results*. A total of 57 articles were identified. The present narrative review summarizes the specific risks for the misuse of prescription opioid medication among women who have experienced violence and trauma, Aboriginal women, adolescents and young women, older women, pregnant women, women of a sexual minority, and transwomen.* Discussion*. The majority of the literature is descriptive, with few studies that evaluate approaches and interventions to respond to the issue of chronic pain, trauma, and misuse of prescription opioids among women, particularly vulnerable subgroups of women.* Conclusions*. Trauma-informed and women-centred approaches that address women's vulnerabilities and complex needs require further attention.

## 1. Introduction

Misuse or abuse of prescription medications has been defined as the use of prescription medications in doses that do not meet individual clinical needs, use of prescription medications for an extended period of time, or use of prescription medications for the experience or feelings derived from the medication. National data and various studies from both Canada and the United States (US) have identified women to be at a greater risk for the misuse of prescription opioid medications. Based on the Canadian Alcohol and Drug Use Monitoring Survey data, opioid use is higher among Canadian women ≥15 years of age compared with men (18.3% versus 15.5%) [1]. However, rates of prescription medication abuse in Canada do not differ significantly for women and men. A US study examining prescription drug use differences between women and men found that women were 48% more likely than men to use any prescription drugs and also more likely to be prescribed opioids [2]. In the US, there was a 400% increase in deaths from prescription opioids between 1999 and 2004 [3]. Currently, men are more likely to die from prescription opioid overdose; however, the gender gap is closing [2]. A study involving 29,906 women and men attending US treatment centres found that, in the past month, women reported significantly greater abuse of prescription opioids (15.4% of women versus 11.1% of men; *P* < 0.001) [4]. Women obtained the medications primarily from family, friends, acquaintances, or their own prescriptions, while men were more likely to obtain opioids from dealers. Several reports from the Substance Abuse and Mental Health Services Administration (SAMHSA) in the US noted high rates of prescription opioid misuse among women seeking substance use treatment and other health services. Women and men 18 to 34 years of age reported the highest primary abuse of prescription opioid medications, and 19% of female and 12.2% of male admissions for substance abuse treatment were for prescription opioid medications [5]. In addition, emergency department visits involving the misuse of opioids showed a slightly higher increase between 2004 and 2008 among women (113% increase) than men (110%) [5].

Misuse of prescription drugs is associated with increased resting heart rate, cognitive impairment, mental health issues, organ damage, and potential for overdose and death [6, 7]. Overuse of opioid medications may also lead to chronic headaches, a condition termed medication overuse headache, which primarily affects women [8]. A literature review by Darnall et al. [9] regarding the effects of long-term opioid use among women reveals several sex- and gender-specific risks, including endocrine disruption, infertility, neonatal health risks, and increased risk for anxiety and depression, as well as cardiac health concerns and potential for overdose [9]. Given these patterns of use and health risks, further attention to prescription drug use and chronic pain treatment among women is required.

*Factors Associated with Women's Misuse of Prescription Drugs*. Various sex-based factors may affect women's misuse of prescription opioid drugs. Women have longer life expectancies relative to men and, therefore, may be more likely to experience chronic pain or other health issues associated with prescription drug use [7]. Some evidence suggests that women may be more sensitive to pain than men due to differences in sex hormones, genetics, or the cortical processing of pain [10]. Sex-specific differences have been found in opioid receptors, leading to a slower onset and offset of morphine among women than men and the need for greater doses to achieve similar effects [11]. Women are more likely to experience chronic pain, report greater intensity of pain, and tend to experience more sensitivity to experimental pain stimuli [12]. Due to women's smaller body mass relative to men and differences in the absorption, metabolism, and elimination of certain medications, the therapeutic window for women may be smaller, and they may be more likely to experience dependency and withdrawal [11, 13].

Higher prevalence rates of prescription opioid medication use may also be explained by gender-related factors. For example, women make more frequent health care visits than men and are often targeted in pharmaceutical marketing [7]. The sensory experience of pain is shaped not only by neurological processes but also by social and cultural expectations and meanings [14]. The tendency for women to report more pain and more intense pain may be related to greater catastrophization of pain among women. This, in part, reflects gender role socialization which teaches girls early on that it is more acceptable for them to express pain than boys. A study of gender norms and hypothetical and experimental pain tolerance revealed that both women and men believed that men should be able to tolerate greater pain than women [15]. In addition, the extent that women and men identified with gendered social norms (i.e., that the ideal man is masculine and can tolerate more pain, and the ideal woman is feminine and tolerates less pain) correlated with reports of pain. Gender-related factors may mediate the experience of pain and may shape women and men's use of prescription opioids including differences in gender role beliefs, coping mechanisms, mood, and expectations of pain [10, 16, 17].

There is evidence to suggest that increased availability of prescription medications is associated with a greater risk for misuse [4, 18]. Medications with abuse potential are more often prescribed to women [19]. Physicians more often prescribe higher doses of opioids to women for medical use [13]. An observational study investigating emergency department pain management practices in 16 US and three Canadian hospitals found that physician gender was also associated with pain management decisions [20]. Female patients were more likely to be prescribed opioids. Female physicians were more likely to prescribe opioids to female patients and male physicians to male patients. Women misusing prescription drugs may also perceive these drugs to be safer and less stigmatized compared with illicit drugs [21, 22]. Due, in part, to these factors affecting prescribing patterns, women ingest more medications than men and, therefore, may be more likely to experience adverse affects [11].

Given the current trends in prevalence of use and prescribing practices and the complex risk factors associated with misuse, prescription opioid drug use and misuse among women and girls is of particular concern. In response, we conducted a scoping review as part of a larger research project on the intersections of chronic pain, experiences of trauma, and misuse of prescription opioid drugs by girls and women.

## 2. Methods

To investigate issues surrounding prescription opioid drug use, trauma, and noncancer chronic pain among women, a scoping review examining sex- and gender-based risks and issues and treatment considerations for a range of subgroups of women who experience chronic noncancer pain and trauma was performed. An environmental scan involving 37 treatment providers, practitioners, community advocates, researchers, policy makers, or medical school instructors to identify priority research and practice issues implicated in girls' and women's misuse of prescription opioid medications was also performed. Finally, face-to-face and online consultations were conducted with researchers, treatment providers, and community partners to present and discuss findings from the environmental scan and scoping review and to identify key issues, priorities, and recommendations. The present review reports on findings from the scoping review only.

An academic database search was conducted for articles published between January 1990 and May 2014. Databases included the following: Academic Search Complete; Bibliography of Native North Americans; Cumulative Index to Nursing and Allied Health Literature (CINAHL); Education Resources Information Centre (ERIC); Family and Society Studies Worldwide; Lesbian, Gay, Bisexual, and Transgender (LGBT) Life with Full Text; MEDLINE; PsycINFO; Social Work Abstracts; and Women's Studies International. A description of the search terms used is provided in Supplementary Material (Appendix 1) available online at http://dx.doi.org/10.1155/2016/1754195. All searches were limited to articles published in the English language and with human subjects. A total of 15 relevant articles were retrieved. Additionally, 22 references were located by individually searching journals using the search terms. The journals searched included Pain Research and Management; The Journal of Pain; The Journal of Pain Research; and Pain Medicine. Five more references were included from reference searching particularly relevant journal articles. The grey literature was also searched for relevant materials. A list of websites and search terms used is provided in Supplementary Material (Appendix 1). In total, 23 relevant papers were identified in the website searches. In addition, Google (Google Inc., US) searches identified another 43 references. Note that 23 papers were excluded after review that did not include information regarding women or differences between women and men (Supplementary Material, Appendix 2). A total of 57 papers with findings relevant to women's chronic noncancer pain and prescription opioid medication misuse are reported narratively in the following section.

## 3. Results

Although few nondrug interventions and programs were identified to address chronic pain and prescription medication misuse, particularly for at-risk subgroups of women, the available evidence regarding risks, issues, and treatment considerations for subgroups of women who experience chronic noncancer pain and trauma is presented.

### 3.1. Women with Trauma Histories

Women are more likely than men to experience trauma, gender-based violence, and psychological effects of trauma, such as anxiety and depression, which may increase their vulnerability to prescription opioid use and misuse [23]. Women who have experienced trauma are more likely to report experiencing unrecognized or difficult to treat medical conditions such as fibromyalgia [24], irritable bowel syndrome, chronic fatigue syndrome, and premenstrual syndrome [4] and may use prescription opioid medications to cope with trauma and associated health effects. Women who have experienced trauma, including intimate partner violence (IPV) and physical and sexual abuse, exhibit numerous physical health problems including physical injuries, chronic health issues, and stress- and mental health-related issues, including depression, anxiety, and posttraumatic stress disorder (PTSD) [25], and are at a greater risk for misusing prescription medications [26]. High rates of trauma have been reported in the US among individuals prescribed opioids for chronic pain, particularly women who report high rates of sexual abuse, physical abuse, violence, or mental health issues [27–29]. A longitudinal study of women in Norway who had experienced IPV revealed that these women received prescriptions for addictive medications (including opioid analgesics) more often compared with women who had not experienced IPV [30].

Multiple studies provide evidence to support the link between trauma, chronic pain, and risk for prescription medication misuse. Women who have experienced abuse and violence are more likely to experience chronic pain. A study involving female survivors of IPV revealed that women who experienced IPV were at greater risk for chronic pain, even in the absence of physical assault [31]. In addition, women who had experienced lifetime abuse-related injury or childhood abuse were also more likely to experience chronic pain. Similarly, a study involving primarily Caucasian women in New England (US) found that women who reported domestic or child abuse were more likely to report chronic pain than women in the control group [32]. A study involving female veterans in the US (*n* = 213) found that the majority of women reported chronic pain (78%) and that a history of sexual trauma was associated with greater severity of pain [33]. In a descriptive study involving women who had separated from an abusive partner, over one-third reported high disability pain [34]. Women reporting high disability pain were more likely to be using more than the prescribed dosages of opioids and nonsteroidal anti-inflammatory medications. High disability pain was also associated with sexual abuse, child abuse, depression, PTSD, suicide attempts, insomnia, unemployment, and medical visits. Finally, one study found that psychological abuse severity and chronic pain severity were mediated by PTSD symptom severity in a sample of Chinese women [35].

Other studies reinforce the connection among prescription drug use, mental illness, and trauma among women. A study involving college students found that lifetime major depression and a history of PTSD were associated with increased nonmedical use of prescription drugs (including opioids, tranquilizers, and sedatives), which was defined as using their own prescribed medicines beyond the dose recommended for a use other than what it was prescribed for or sourcing the medication from someone else [36]. Prescription opioid medications are often used concurrently with other medications. For example, a Norwegian study [37] found that previous benzodiazepine use was a greater predictor of opioid use among women who reported chronic pain. While this study measured prescription frequencies, the authors noted that the combined use of these two medications may increase the risk for addictive potential or abuse. Another study involving college students (*n* = 22,783; *n* = 15,780 women, *n* = 6913 men) found that opioid misuse (defined as use without a prescription, although no details were provided regarding where medications were sourced) was higher for women than men (66.5% versus 34.5%, resp.) and that depressive symptoms and suicidality were significantly associated with greater odds for prescription opioid misuse [38]. Women who reported feeling sad or depressed or who had attempted suicide had between 1.26 and 1.32 greater odds of reporting opioid misuse. A qualitative study examining the use of opioids and benzodiazepines by female illicit (methamphetamine) drug users found that the use of these prescription medications was often initiated to cope with trauma and chronic pain and that some women switched to prescription opioids due to easier accessibility [22]. Women reported finding it easier to obtain prescription opioids compared with methamphetamine, either through their own prescription or through the prescriptions of their partners or friends.

### 3.2. Aboriginal and Indigenous Women

Prescription drug use, particularly opioid use, is high among First Nations and Inuit people in Canada. Data from the Noninsured Health Benefits Program revealed that in Ontario, in 2007, 898 opioid prescriptions were prescribed per 1000 First Nations individuals ≥15 years of age and, in response to this change, have since been made to improve prescription monitoring to reduce the risk for abuse [7]. In Canada, prescription medication use rates are reportedly higher among Aboriginal women than Aboriginal men [39]. In a commentary, Webster [40] reports that opioid addiction among Aboriginals often can be linked to overprescribing by physicians (often on short-term contracts in Aboriginal communities); once individuals become addicted and if they can no longer find a physician to refill their prescriptions, they seek out illegal sources for the medications. Similarly, in the US, a qualitative study exploring oxycontin (an opioid pain-killer) use among American Indians found that participants reported an increase in oxycontin use on their reservation as well as other reservations, with use being the highest among women 19 to 38 years of age [41]. Reported sources of oxycontin include prescriptions from doctors, family, and friends or buying pills from community members. High rates of trauma, gendered and colonial violence, poor health status, and poverty are all factors implicated in high rates of prescription opioid use among Aboriginal populations [42]. A US report noted that American Indians and Alaskan Natives have unique experiences that may hinder pain management, including cultural and geographical barriers, low income, and poor living conditions [43]. These groups may also have distinct perspectives related to pain management; for example, there is some evidence that American Indian/Alaskan Native patients experiencing chronic pain may underemphasize or not want to discuss their experience of pain. Based on the hypothesis that rural and remote Aboriginal communities may be somewhat protected against substance use problems through cultural participation, one study examined the links between illicit and prescription drug abuse (including, but not limited to, opioid misuse) and Aboriginal enculturation among urban Aboriginals in a Canadian city [44]. They found that Aboriginal cultural involvement was protective of prescription drug abuse within this particular urban setting. Surveys revealed that individuals who reported cultural participation scored higher on self-esteem measures. Conversely, mainstream acculturation was a risk factor for prescription drug misuse. While the authors do not discuss gender differences, over one-half (58.6%) of the participants were female.

### 3.3. Pregnant Women

Prescription medication misuse among pregnant women is also a significant problem affecting both women's health and fetal health and increases the risk for neonatal abstinence syndrome [45]. In the US, neonatal abstinence syndrome rose by 300% between 2000 and 2009 [3]. Some research has reported that prescription medication borrowing and sharing are higher (36.5%) among women of reproductive age (18 to 44 years of age) than any other group (total *n* = 26,566), with opioids being one of the prescriptions most commonly shared [46]. High rates of prescription opioid medication use during pregnancy have been reported among Aboriginal women. A 2011 survey [40] found that 17% of pregnant Aboriginal women in Northwestern Ontario misused opioid medications. Pregnant women using prescription opioid medications during pregnancy often use other substances concurrently, including alcohol and tobacco, creating additional health risks for the mother and the fetus [47, 48].

### 3.4. Medical Use of Opioids and Opioid Management during Pregnancy

Several reports provide suggestions regarding opioid use and reproduction among women. The Canadian Guideline For Safe and Effective Use of Opioids For Chronic Noncancer Pain notes the need for caution in opioid use among pregnant and breastfeeding women but does not include other sex- or gender-specific guidelines [49]. A report from the British Pain Society notes that prolonged opioid use can result in amenorrhoea, fertility issues, and depression in women, and patients should be advised of these potential issues before commencing opioid use [50]. They also note that women who are pregnant may need to be engaged in alternate forms of pain management due to the possible fetal health effects (particularly neonatal withdrawal). A report from Ontario addressing prescription medication misuse notes the need for increased management of pregnant women addicted to opioids (in particular, improving access to other safer medications) [51].

### 3.5. Young Women

Several studies have reported high rates of misuse of prescription drugs among adolescents and young women. Canadian Alcohol and Drug Use Monitoring Survey data revealed an increase in the use of psychoactive medications (including opioids, stimulants, and sedatives) between 2011 (17.6%) and 2012 (24.7%) among youth, with a higher prevalence in 2012 among females (26.7%) compared with males (21.3%) [1]. A descriptive study examined the misuse of prescription opioids among students 10 to 18 years of age in Detroit (US) (*n* = 1017; female = 508, male = 509) [52]. A total of 22% of girls and 10% of boys reported misuse of medication in their lifetime, and 15% of girls and 7% of boys had used prescription opioids in the past year. Students who reported misusing prescription opioids were more likely to smoke cigarettes and marijuana and drink alcohol. The primary method of obtaining medications was from a family member (24%). Analysis of a survey conducted among students (grades seven to 12, *n* = 2914, 49.4% male, 50.6% female) in Ontario schools in 2007 found that female students were more likely to have used an opioid analgesic in the past year either medically or nonmedically, although there were no differences in using opioids only nonmedically [53]. Similar to the previous study, the majority of students reported sourcing medications in their homes (72%). A 2011/2012 survey in the US examining opioid use among adolescents (*n* = 2964; 51% female, 49% male) found that females who had been prescribed opioids were more likely to report misuse (using more than prescribed or using it for nonmedical reasons) than males (21.8% versus 11.2%) [54]. Finally, a survey of emergency department visits related to opioid misuse among adolescents in 2011 revealed similar admittance rates for males and females; however, admittance for certain opioid medications (including oxycodone and hydromorphone) tripled between 2005 and 2011 among females [55].

Other studies have reported somewhat higher rates of use or misuse among boys and young men than girls or no differences at all. A 2005 survey by SAMSHA revealed that past year prescription opioid misuse among young individuals 18 to 25 years of age was slightly higher among males (13.5%) than females (11.3%) [56]. Young women were more likely to have sourced opioids from a friend or relative (58.9% compared with 48.2% among men). Finally, a national survey involving US high school senior students (*n* = 7374; 48% female, 52% male) found that 12.9% reported misuse of opioids; they did not observe differences between males and females [57]. The majority of participants (80%) reported obtaining the prescription opioid from a previous medical prescription.

### 3.6. Older Women

Several reports note the greater risk for opioid misuse among older women compared with older men. Based on SAMSHA data from the US, among individuals >65 years of age admitted to substance abuse treatment, women reported three times greater abuse of opioid medications than men [58]. Those more at risk for psychoactive prescription medication abuse (including opioids) include women who are older, those with a history of substance abuse or mental health concerns, and those who are socially isolated [58]. Greater use and misuse of prescription medications among older women may be connected to the loss of a partner, low income, mental health issues, or poor overall health. A systematic review revealed that the use of opioids and benzodiazepines contributes to, and increase the risk for, cognitive deficits among older adults [59].

### 3.7. Lesbian, Bisexual, Two-Spirited, and Transgendered Individuals

Several studies and reports note factors which increase vulnerability among lesbian, bisexual, two-spirited (a term used by First Nations people to describe a person who identifies as having both a feminine and masculine spirit), and transgendered individuals to use and misuse opioid drugs. For example, lesbians are at higher risk for trauma and abuse than heterosexual women and may experience PTSD following sexual abuse as well as homophobic violence [60]. Compared with heterosexual women, lesbians and bisexual women are more than twice as likely to report lifetime victimization (including partner abuse, childhood abuse, physical violence, and sexual assault) and report more victimization experiences [61]. Lesbian, gay, bisexual, transgender, or queer (LGBTQ) individuals are more likely to experience psychological distress, discrimination, depression or anxiety, and substance use and to report greater mental health needs [62]. LGBTQ individuals are also more likely to experience barriers in accessing employment and housing and health services, as well as more bullying and abuse, and a lack of services and social programs that are culturally appropriate [63].

## 4. Discussion

Overall, there is a dearth of studies that describe or evaluate approaches and interventions to respond to the issue of chronic pain, trauma, and misuse of opioid drugs among women and particularly vulnerable subgroups of women. The vast majority of articles identified in the present scoping review were descriptive in nature. While the problem of opioid misuse among women is clear, there are few interventions that have addressed this issue among women and vulnerable subgroups of women with a sex- and gender-informed approach. Overall, women-centred, trauma-informed, and harm reduction approaches are lacking. Key principles of women-centred care include equity, empowerment, partnership, self-determination, and respect [64]. Women-centred approaches acknowledge the realities of women's lives including the links between substance use, violence, and abuse [65]. This approach recognizes the potential for shame and stigma and seeks to empower women by providing a safe, respectful, and nonjudgmental environment to facilitate sharing and healing among women [64, 65].

In general, there is also a lack of integrated approaches that recognize the cooccurring nature of trauma, chronic pain, and opioid misuse or the need for recognizing interacting factors. For example, approaches that integrate Indigenous healing practices with trauma-informed care principles to address prescription drug misuse among women are clearly needed. There has been some research exploring the integration of Indigenous healing practices with Western approaches to address substance use among Indigenous youth [66–68] and for Indigenous adults in addictions treatment [69]. While these approaches have not been developed and evaluated for women who use prescription opioids, integrating a more holistic approach to health and healing that addresses physical, spiritual, emotional, social, and mental well-being is justified.

Some reports also highlight the need for integrating consideration of trauma within substance use treatment. Psychosocial stress can cause changes in and over activation of neural structures that contribute to anxiety and chronic pain [70]. Interdisciplinary pain management, which addresses physical pain as well as psychosocial issues and psychological health, brings together a range of practitioners (physician, psychologists, physical therapist, nurse, pharmacist, etc.) to tailor a treatment plan to meet individual needs [70]. These individualized plans could include pharmacological intervention, brief education, and more intensive rehabilitation programs (including, e.g., physical therapy and cognitive behavioral therapies).

Several reports note strategies for preventing prescription drug misuse, including those that address screening and prescribing practices of physicians as well as health promotion initiatives. Guidelines are required to assist providers in minimizing addiction to opioids, identifying and managing addiction, and offering alternatives to opioids [71]. This is particularly relevant given that the majority of articles reviewed reveal that women and girls tend to obtain prescription opioids from their own prescriptions or from those of family and friends. Screening can be used to identify patients who are not responsive to initial pain management measures [72]. Approaches to screening that are gender-informed, trauma-informed, and culturally sensitive are being identified in the substance use literature overall [73, 74]. Improved health promotion, social marketing, and product information, as well as instituting risk reduction programs for individuals, are among the recommendations from the Canadian Centre on Substance Abuse. In particular, the centre suggests that women comprise a population at risk in need of tailored approaches to this problem [7].

Also, improved knowledge translation is required which engages researchers and various knowledge users in synthesizing, exchanging, and applying information related to the treatment of chronic pain [75]. Henry [75] notes that the need for improvements in care for chronic pain is widely documented in the literature but that an improved process is needed for engaging stakeholders and developing recommendations; he proposes a national program which engages researchers and the community to address chronic pain. Within knowledge translation efforts, understanding can be promoted of sex and gendered influences, factors related to chronic pain, experience of violence, trauma, and prescription drug use, as well as gender-informed prevention, harm reduction, and treatment approaches.

## 5. Conclusions

There are clearly specific risks for pain and the misuse of prescription opioid medication among subgroups of women including those who have experienced violence and trauma, Aboriginal women, adolescents and young women, older women, pregnant women, women of a sexual minority, and transwomen. The available literature suggests that these groups have specific vulnerabilities and complex intervention needs that require further attention, both in prevention and in response. Clearly, the misuse of opioid medications among women is a significant issue, complicated by a multitude of factors including but not limited to sex and gender differences in pain and gender-related factors affecting prescribing practices, trauma, mental health, ethnicity, substance use, and psychosocial factors. Evidence-based interventions to address prescription opioid misuse among these subgroups are lacking or limited, despite these features and predisposing factors. Given the range of characteristics, and the development and evaluation of trauma-informed, women-centred interventions, approaches that are sex- and gender-specific to address these complex and often interrelated factors are necessary to effectively manage women's pain and support mental wellness.

## Supplementary Material

A list of the academic literature search terms, a list of grey literature search terms and the websites searched, and a list of references that were captured by the searches but excluded from the final review due to lack of relevance.

## References

[1] Health Canada Canadian Alcohol and Drug Use Monitoring Survey: Summary of Results for 2012. http://www.hc-sc.gc.ca/hc-ps/tobac-tabac/research-recherche/stat/ctums-esutc_2012-eng.php.

[2] Simoni-Wastila L. (2000). The use of abusable prescription drugs: the role of gender. *Journal of Women's Health and Gender-Based Medicine*.

[3] Centers for Disease Control and Prevention (1999). Vital signs: overdoses of prescription opioid pain reliever, United States, 1999–2008. *Morbidity and Mortality Weekly Report (MMWR)*.

[4] Green T. C., Grimes Serrano J. M., Licari A., Budman S. H., Butler S. F. (2009). Women who abuse prescription opioids: findings from the Addiction Severity Index-Multimedia Version® Connect prescription opioid database. *Drug and Alcohol Dependence*.

[5] Crane E. H. (2015). *The CBHSQ Report: Emergency Department Visits Involving Narcotic Pain Relievers*.

[6] Kelly B. C., Wells B. E., LeClair A., Tracy D., Parsons J. T., Golub S. A. (2013). Prevalence and correlates of prescription drug misuse among socially active young adults. *International Journal of Drug Policy*.

[7] Canadian Centre on Substance Abuse. National Advisory Committee on Prescription Drug Misuse (2013). *First Do No Harm: Responding to Canada's Prescription Drug Crisis*.

[8] Katsarava Z., Obermann M. (2013). Medication overuse headache. *Top Pain Management*.

[9] Darnall B. D., Stacey B. R., Chou R. (2012). Medical and psychological risks and consequences of long-term opioid therapy in women. *Pain Medicine*.

[10] Keogh E. (2006). Sex and gender differences in pain: a selective review of biological and psychosocial factors. *Journal of Men's Health and Gender*.

[11] Soldin O. P., Chung S. H., Mattison D. R. (2011). Sex differences in drug disposition. *Journal of Biomedicine and Biotechnology*.

[12] Foreman J. (2014). Why women are living in the discomfort zone. *The Wall Street Journal*.

[13] Kelly-Blake K. (2013). Overdosing on prescription painkillers: dying for pain relief?. *Bioethics in the News*.

[14] Einstein G. (2007). *Sex and the Brain: A Reader*.

[15] Pool G. J., Schwegler A. F., Theodore B. R., Fuchs P. N. (2007). Role of gender norms and group identification on hypothetical and experimental pain tolerance. *Pain*.

[16] Hurley R. W., Adams M. C. B. (2008). Sex, gender, and pain: an overview of a complex field. *Anesthesia and Analgesia*.

[17] Greenspan J. D., Craft R. M., LeResche L. (2007). Studying sex and gender differences in pain and analgesia: a consensus report. *Pain*.

[18] Homish G. G., Leonard K. E., Cornelius J. R. (2010). Individual, partner and relationship factors associated with non-medical use of prescription drugs. *Addiction*.

[19] Cook D. A. (2007). *Addiction and Medications*.

[20] Safdar B., Heins A., Homel P. (2009). Impact of physician and patient gender on pain management in the emergency department—a multicenter study. *Pain Medicine*.

[21] Tuchman E. (2010). Women and addiction: the importance of gender issues in substance abuse research. *Journal of Addictive Diseases*.

[22] Lamonica A., Boeri M. (2012). An exploration of the relationship between the use of methamphetamine and prescription drugs. *Journal of Ethnographic & Qualitative Research*.

[23] United Nations Office on Drugs and Crime (2011). *The Non-Medical Use of Prescription Drugs: Policy Direction Issues*.

[24] Shuster J., McCormack J., Riddell R. P., Toplak M. E. (2009). Understanding the psychosocial profile of women with fibromyalgia syndrome. *Pain Research and Management*.

[25] Cole J., Logan T. K. (2010). Nonmedical use of sedative-hypnotics and opiates among rural and urban women with protective orders. *Journal of Addictive Diseases*.

[26] McCauley J. L., Amstadter A. B., Danielson C. K., Ruggiero K. J., Kilpatrick D. G., Resnick H. S. (2009). Mental health and rape history in relation to non-medical use of prescription drugs in a national sample of women. *Addictive Behaviors*.

[27] Balousek S., Plane M. B., Fleming M. (2007). Prevalence of interpersonal abuse in primary care patients prescribed opioids for chronic pain. *Journal of General Internal Medicine*.

[28] Hart-Johnson T., Green C. R. (2012). The impact of sexual or physical abuse history on pain-related outcomes among blacks and whites with chronic pain: gender influence. *Pain Medicine*.

[29] Jamison R. N., Butler S. F., Budman S. H., Edwards R. R., Wasan A. D. (2010). Gender differences in risk factors for aberrant prescription opioid use. *Journal of Pain*.

[30] Stene L. E., Dyb G., Tverdal A., Jacobsen G. W., Schei B. (2012). Intimate partner violence and prescription of potentially addictive drugs: prospective cohort study of women in the Oslo Health Study. *BMJ Open*.

[31] Wuest J., Ford-Gilboe M., Merritt-Gray M. (2010). Pathways of chronic pain in survivors of intimate partner violence. *Journal of Women's Health*.

[32] Kendall-Tackett K., Marshall R., Ness K. (2003). Chronic pain syndromes and violence against women. *Women and Therapy*.

[33] Haskell S. G., Papas R. K., Heapy A., Reid M. C., Kerns R. D. (2008). The association of sexual trauma with persistent pain in a sample of women veterans receiving primary care. *Pain Medicine*.

[34] Wuest J., Merritt-Gray M., Ford-Gilboe M., Lent B., Varcoe C., Campbell J. C. (2008). Chronic pain in women survivors of intimate partner violence. *Journal of Pain*.

[35] Tiwari A., Fong D. Y. T., Chan C.-H., Ho P.-C. (2013). Factors mediating the relationship between intimate partner violence and chronic pain in Chinese women. *Journal of Interpersonal Violence*.

[36] McCauley J. L., Amstadter A. B., Macdonald A. (2011). Non-medical use of prescription drugs in a national sample of college women. *Addictive Behaviors*.

[37] Skurtveit S., Furu K., Bramness J., Selmer R., Tverdal A. (2010). Benzodiazepines predict use of opioids—a follow-up study of 17,074 men and women. *Pain Medicine*.

[38] Zullig K. J., Divin A. L. (2012). The association between non-medical prescription drug use, depressive symptoms, and suicidality among college students. *Addictive Behaviors*.

[39] First Nations and Inuit Health. Prescription Drug Abuse (PDA) Update (2012). *Presentation to the NNADAP Renewal Leadership Team*.

[40] Webster P. C. (2013). Indigenous Canadians confront prescription opioid misuse. *The Lancet*.

[41] Momper S. L., Delva J., Reed B. G. (2011). OxyContin misuse on a reservation: qualitative reports by American Indians in talking circles. *Substance Use and Misuse*.

[42] Dell C. A., Roberts G., Kilty J. (2012). Researching prescription drug misuse among First Nations in Canada: starting from a health promotion framework. *Substance Abuse: Research and Treatment*.

[43] http://www.inthefaceofpain.org/.

[44] Currie C. L., Wild T. C., Schopflocher D. P., Laing L., Veugelers P. (2013). Illicit and prescription drug problems among urban Aboriginal adults in Canada: the role of traditional culture in protection and resilience. *Social Science and Medicine*.

[45] Dryden C., Young D., Hepburn M., Mactier H. (2009). Maternal methadone use in pregnancy: factors associated with the development of neonatal abstinence syndrome and implications for healthcare resources. *BJOG: An International Journal of Obstetrics and Gynaecology*.

[46] Petersen E. E., Rasmussen S. A., Daniel K. L., Yazdy M. M., Honein M. A. (2008). Prescription medication borrowing and sharing among women of reproductive age. *Journal of Women's Health*.

[47] Winklbaur B., Kopf N., Ebner N., Jung E., Thau K., Fischer G. (2008). Treating pregnant women dependent on opioids is not the same as treating pregnancy and opioid dependence: a knowledge synthesis for better treatment for women and neonates. *Addiction*.

[48] Greaves L., Poole N., Okoli C. T. (2011). *Expecting to Quit: A Best Practices Review of Smoking Cessation Interventions for Pregnant and Post-Partum Women*.

[49] National Opioid Use Guideline Group (2010). *Canadian Guideline for Safe and Effective Use of Opioids for Chronic Non-Cancer Pain. Part B: Recommendations for Practice*.

[50] The British Pain Society (2010). *Opioids for Persistent Pain: Good Practice*.

[51] Expert Working Group on Narcotic Addiction (2015). *The Way Forward: Stewardship for Prescription Narcotics in Ontario 2012*.

[52] Boyd C. J., Esteban McCabe S., Teter C. J. (2006). Medical and nonmedical use of prescription pain medication by youth in a Detroit-area public school district. *Drug and Alcohol Dependence*.

[53] Brands B., Paglia-Boak A., Sproule B. A., Leslie K., Adlaf E. M. (2010). Nonmedical use of opioid analgesics among Ontario students. *Canadian Family Physician*.

[54] McCabe S. E., West B. T., Boyd C. J. (2013). Motives for medical misuse of prescription opioids among adolescents. *Journal of Pain*.

[55] SAMHSA (2012). Emergency department visits by adolescents involving narcotic pain relievers. *The DAWN Report*.

[56] SAMHSA (2006). *How Young Adults Obtain Prescription Pain Relievers for Nonmedical Use*.

[57] McCabe S. E., West B. T., Teter C. J., Boyd C. J. (2012). Medical and nonmedical use of prescription opioids among high school seniors in the United States. *Archives of Pediatrics and Adolescent Medicine*.

[58] SAMHSA (2012). *Issue Brief 5: Prescription Medication Misuse and Abuse among Older Adults*.

[59] Tannenbaum C., Paquette A., Hilmer S., Holroyd-Leduc J., Carnahan R. (2012). A systematic review of amnestic and non-amnestic mild cognitive impairment induced by anticholinergic, antihistamine, GABAergic and opioid drugs. *Drugs and Aging*.

[60] Ziegler P. (2010). Addiction and chronic pain in lesbians. *Journal of Gay and Lesbian Mental Health*.

[61] Hughes T., McCabe S. E., Wilsnack S. C., West B. T., Boyd C. J. (2010). Victimization and substance use disorders in a national sample of heterosexual and sexual minority women and men. *Addiction*.

[62] Lamoureux A., Joseph A. J. (2014). Toward transformative practice: facilitating access and barrier-free services with LGBTTIQQ2SA populations. *Social Work in Mental Health*.

[63] Gay and Lesbian Medical Association Healthy people 2010: Companion document for lesbian, gay, bisexual, and transgender (LGBT) health. http://www.med.umich.edu/diversity/pdffiles/healthpeople.pdf.

[64] Cory J. (2007). *Women-Centred Care: A Curriculum for Health Care Providers*.

[65] Covington S. S. (2008). Women and addiction: a trauma-informed approach. *Journal of Psychoactive Drugs*.

[66] Smylie J., Kaplan-Myrth N., McShane K., Nation P. F. (2009). Indigenous knowledge translation: baseline findings in a qualitative study of the pathways of health knowledge in three indigenous communities in Canada. *Health Promotion Practice*.

[67] Dell C. A., Seguin M., Hopkins C. (2011). From benzos to berries: treatment offered at an aboriginal youth solvent abuse treatment centre relays the importance of culture. *Canadian Journal of Psychiatry*.

[68] Dell D., Hopkins C. (2011). Residential volatile substance misuse treatment for indigenous youth in Canada. *Substance Use and Misuse*.

[69] Rowan M., Poole N., Shea B. (2014). Cultural interventions to treat addictions in indigenous populations: findings from a scoping study. *Substance Abuse: Treatment, Prevention, and Policy*.

[70] Meagher M. W., Kendall-Tackett K. (2004). Links between chronic pain and traumatic family violence: biopsychosocial pathways and clinical implications. *Health Consequences of Abuse in the Family: A Clinical Guide for Evidence-Based Practice*.

[71] Compton W. M., Volkow N. D. (2006). Major increases in opioid analgesic abuse in the United States: concerns and strategies. *Drug and Alcohol Dependence*.

[72] California Code of Regulations (2009). Chronic pain medical treatment guidelines. *Medical Treatment Utilization Schedule*.

[73] Poole N., Urquhart C., Jasiura F., Smylie D., Schmidt R. (2013). *Trauma-Informed Practice Guide*.

[74] SAMHSA (2014). *Trauma-Informed Care in Behavioral Health Services: Treatment Improvement Protocol (TIP)*.

[75] Henry J. L. (2008). The need for knowledge translation in chronic pain. *Pain Research and Management*.

