# Supplementary material for: Misuse of Prescription Opioid Medication among Women: A Scoping Review
Source: Pain Res Manag. 2016 Apr 17;2016:1754195. doi: 10.1155/2016/1754195 (PMC4904621; doi:10.1155/2016/1754195)
Supplement: Supplementary file 1 — A list of the academic literature search terms, a list of grey literature search terms and the websites searched, and a list of references that were captured by the searches but excluded from the final review due to lack of relevance. [file 1754195.f1.pdf]

Appendix 1: Academic Database & Grey Literature Search Terms

Searches of each Academic database were conducted using the following search terms: 1) women + trauma + chronic pain; 2) women + trauma + pain medication; 3) women + trauma + medication; 4) women + trauma + prescription pain relie*; 5) women + trauma + prescription drug use, 6) women + trauma + opiate; 7) women + trauma + opioid; 8) women + abuse + opioid*; 9) women + prescription drug misuse; 10) girls + chronic pain; 11) girls + prescription drug misuse; 12) girls + opioid*; 13) Aboriginal OR First Nation* + chronic pain; 14) Aboriginal OR First Nation* + prescription drug misuse; 15) Aboriginal OR First Nation* + opioid*; 16) prescription + seeking + women; 17) opioid* + prescribing practice.

Google searches were conducted using the following search terms: 1) Canadian opioid guidelines; 2) non medical use of prescription pain relievers; 3) pain relievers + trauma + women; 4) childhood trauma + chronic pain + opioid; 5) childhood trauma + chronic pain + opiates; 6) abuse + pain relievers + women; 6) abuse + pain prescription + women; 7) abuse + opioids + women; 8) intimate partner violence + opioids + women; 9) domestic violence + opioids + women; 10) intimate partner violence + prescription + women; 11) PTSD + opioids + women; 12) pain medication+ Aboriginal + women; 13) prescription pain relievers+ Aboriginal + women; 14) prescription pain relief + First Nations; 15) pain medication + First Nations; 16) opioid + First Nations;17) prescription drug + abuse + First Nation; 18) prescription pain relief + adolescent girls; 19) pain medication + adolescent girls; 20) opioid + adolescent girls; 21) prescription drug + abuse + adolescent girls; 22) prescription pain relief + patient seeking behavior; 23) opioid + patient seeking behavior; 24) opioid + patient seeking behavior + women; 25) narcotic + patient seeking behavior + women.

The following websites were identified during the grey literature search, and searched for

relevant references:

- ●
- Substance Abuse and Mental Health Services Administration (http://www.samhsa.gov/)
  - Search terms: pain relievers; pain relievers + women; prescription pain relievers + abuse + women; pain + trauma
- AddictionPain.com
  - Looked up references for the "Trauma and Pain" page
- National Centre for Complementary and Alternative Medicine (http://nccam.nih.gov/)
  - Search terms: chronic pain; pain + women; trauma; addiction; prescription
- Addiction Free Pain Management (http://www.addiction-free.com/)
  - Scanned "Publications" page
- American Chronic Pain Association (http://www.theacpa.org/)
  - Search terms: women; trauma; pain relievers + women; prescription pain relievers + abuse + women; pain + trauma
- American Academy of Pain Management (http://www.aapainmanage.org/)
  - Scanned "Publications" and "Resources" pages
- Pain Pathways (http://www.painpathways.org/)
  - Search terms: women; trauma; abuse; opioid; pain relievers + women; prescription pain relievers + abuse + women; pain + trauma
- American Pain Foundation (http://www.painfoundation.org/)
- American Pain Society (http://www.americanpainsociety.org/)
  - Scanned "Publications, Resources and Guidelines" page
- American Society of Interventional Pain Physicians (http://www.asipp.org/)
  - Scanned "Publications and Guidelines" page

Appendix 2: Located Studies with no information on Sex or Gender

The following studies were reviewed, but excluded because they did not include any information about women or gender:

1. Asmundson, G.J.G., et al., PTSD and the Experience of Pain: Research and Clinical Implications of Shared Vulnerability and Mutual Maintenance Models. *Canadian Journal of Psychiatry*, 2002. 47(10): p. 930.
2. Becker, W.C., et al., The Association Between Chronic Pain and Prescription Drug Abuse in Veterans. *Pain Medicine*, 2009. 10(3): p. 531-536.

3. Birnbaum, H.G., et al., Societal Costs of Prescription Opioid Abuse, Dependence, and Misuse in the United States. *Pain Medicine*, 2011. 12(4): p. 657-667.
4. Colameco, S., Opiate Prescribing for Chronic Pain: Minimizing the Risk of Abuse, Dependence, and Diversion. *Perspectives,* 2004. 4(5): p. 13-18.
5. Currie, C.L., et al., *Illicit and prescription drug problems among urban Aboriginal adults in Canada: The role of traditional culture in protection and resilience.* Social Science & Medicine, 2013. **88**(0): p. 1-9.
6. Delgado, R., et al., Assessing the Quality, Efficacy, and Effectiveness of the Current Evidence Base of Active Self-Care Complementary and Integrative Medicine Therapies for the Management of Chronic Pain: A Rapid Evidence Assessment of the Literature. *Pain Medicine*, 2014. 15: p. S9-S20.
7. Fibbi, M., et al., Denial of Prescription Opioids Among Young Adults with Histories of Opioid Misuse. *Pain Medicine*, 2012. 13(8): p. 1040-1048.
8. Ghisi, M., et al., *Psychological Distress and Post-Traumatic Symptoms Following Occupational Accidents.* Behavioral Sciences, 2013. **3**(4): p. 587-600.
9. Hansen, G.R., The Drug-Seeking Patient in the Emergency Room. *Emergency Medicine Clinics of North America,* 2005. 23: p. 349-365.
10. In the Face of Pain, *Race and Pain*, in *Fact Sheet*. 2011, In the Face of Pain: Stamford, CT.
11. Jimenez, N., et al., *A Review of the Experience, Epidemiology, and Management of Pain among American Indian, Alaska Native, and Aboriginal Canadian Peoples.* The Journal of Pain, 2011. **12**(5): p. 511-522.

12. Kahan, M., A. Mailis-Gagnon, and E. Tunks, Canadian Guideline For Safe and Effective Use of Opioids for Chronic Non-Cancer Pain: Implications for pain physicians. *Pain Research and Management,* 2010. 16(3): p. 157-158.
13. Lidel, A., et al., Support for the mutual maintenance of pain and post-traumatic stress disorder symptoms. *Psychological Medicine,* 2010. 40: p. 1215-1223.
14. Nuckols, T.K., et al., Opioid Prescribing: A Systematic Review and Critical Appraisal of Guidelines for Chronic Pain. *Annals of Internal Medicine*, 2014. 160(1): p. 38-47.
15. New South Wales Health, *Responsible opioid prescribing: identifying and handling drug-seeking patients,* 2008, New South Wales Health: North Sydney, Australia
16. Office of National Drug Control Policy, *Prescription for Danger: A report on the troubling trend of prescription and over-the-counter drug abuse among the nation's teens,* 2008, Office of National Drug Control Policy: Washington, DC.
17. Phillips, J., Prescription drug abuse: Problem, policies, and implications. *Nursing Outlook*, 2013. 61(2): p. 78-84.
18. Prince, V., Pain Management in Patients with Substance-Use Disorders. *Chronic Illnesses,* N.D. VII: p. 171-188.
19. Rieckmann, T., et al., *American Indians with Substance Use Disorders: Treatment Needs and Comorbid Conditions.* The American Journal of Drug and Alcohol Abuse, 2012. **38**(5): p. 498-504.
20. Schieffer, B.M., et al., Pain Medication Beliefs and Medication Misuse in Chronic Pain. *The Journal of Pain,* 2005. 6(9): p. 620-629.

21. Wang, K.H., W.C. Becker, and D.A. Fiellin, *Prevalence and correlates for nonmedical use of prescription opioids among urban and rural residents.* Drug and Alcohol Dependence, 2013. **127**(1–3): p. 156-162.

22. Webster, P.C., *Medically induced opioid addiction reaching alarming levels.* Canadian Medical Association Journal, 2012. **184**(3): p. 285-286.

23. Young, A.M., J.R. Havens, and C.G. Leukefeld, *Route of administration for illicit prescription opioids: a comparison of rural and urban drug users.* Harm reduction journal, 2010. **7**(24): p. 1-7.
